# 860. HIV Post-exposure Prophylaxis Availability at Small and Critical Access Hospitals in the Western Region

**DOI:** 10.1093/ofid/ofab466.1055

**Published:** 2021-12-04

**Authors:** Alyssa Y Castillo, Peter Bulger, John B Lynch, John B Lynch, Paul Pottinger, Carolyn Chu, Jeannie D Chan, Rupali Jain, Mandana Naderi, Zahra Kassamali, Jehan Budak, Jehan Budak, Chloe Bryson-Cahn

**Affiliations:** 1 University of Washington, Seattle, Washington; 2 University of California San Francisco, San Francisco, California; 3 UW Medicine, Harborview Medical Center, Seattle, WA; 4 University of Washington School of Medicine, Seattle, WA; 5 University of Arizona College of Pharmacy, Phoenix, Arizona; 6 UW Medicine, Valley Medical Center, University of Washington, Seattle, WA

## Abstract

**Background:**

Post-exposure prophylaxis (PEP) is essential to minimize the risk of human immunodeficiency virus (HIV) acquisition following an occupational or nonoccupational exposure to potentially infectious body fluids. PEP is most effective when initiated as soon as possible after HIV exposure. Patients in rural areas may rely on small (< 50 beds) and critical access (< 25 beds) hospitals for access to PEP – especially after-hours and on holidays, when outpatient pharmacies are typically closed. However, PEP medications are costly to maintain on a hospital formulary due to unpredictable use and expiration. We hypothesized that PEP availability may be variable and limited at such hospitals.

**Methods:**

The University of Washington Tele-Antimicrobial Stewardship Program (UW-TASP) is comprised of 68 hospitals in Washington, Oregon, Arizona, Idaho, and Utah, most of which are rural and critical access. In August 2020, we surveyed UW-TASP participating hospitals and a convenience sample of other networked rural hospitals in Western states using REDCap, a HIPAA-compliant, electronic data management program. Respondents reported all antimicrobials on their hospital formulary and their hospital size. Data were reviewed by physicians and pharmacists trained in infectious diseases. Preferred PEP regimens, defined by the CDC, for adults and adolescents ≥ 13 years, included combination tenofovir disoproxil fumarate-emtricitabine (TDF/FTC) and either raltegravir (RAL) or dolutegravir (DTG).

**Results:**

Responses from 49 hospitals were received. Six were excluded – one was incomplete and five were excluded due to hospital size ( > 50 beds) (Table 1). The majority of hospitals (40/43, 93.0%) were critical access. Half of the hospitals’ formularies (22/43, 51.2%) contained a preferred PEP regimen. One hospital reported a non-preferred regimen. Most hospitals with a preferred PEP regimen on formulary (18/22, 86.3%) offered TDF/FTC + RAL, and the remainder (4/22, 18.2%) offered TDF/FTC + DTG.

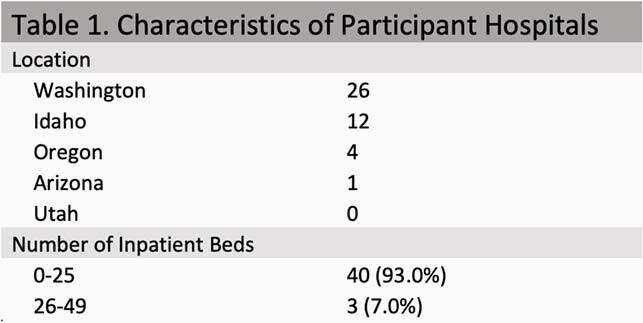

**Conclusion:**

Many small and critical access hospital formularies do not include antiretroviral agents needed for HIV PEP. Improving urgent access to these critical medications in rural communities is an opportunity for HIV prevention.

**Disclosures:**

**Jehan Budak, MD**, Nothing to disclose **Chloe Bryson-Cahn, MD**, **Alaska Airlines** (Other Financial or Material Support, Co-Medical Director, position is through the University of Washington)

